# Possible Missing Sources of Atmospheric Glyoxal Part II: Oxidation of Toluene Derived from the Primary Production of Marine Microorganisms

**DOI:** 10.3390/metabo14110631

**Published:** 2024-11-16

**Authors:** Renee T. Williams, Annika Caspers-Brown, Jennifer Michaud, Natalie Stevens, Michael Meehan, Camille M. Sultana, Christopher Lee, Francesca Malfatti, Yanyan Zhou, Farooq Azam, Kimberly A. Prather, Pieter Dorrestein, Michael D. Burkart, Robert S. Pomeroy

**Affiliations:** 1 Williams Biotech Consulting, San Bruno, CA 90466, USA; rw@ags-tx.com; 2 Department of Chemistry and Biochemistry, University of California, La Jolla, San Diego, CA 92093, USAnatalie.stevens@catalent.com (N.S.); chl257@ucsd.edu (C.L.);; 3 Skaggs School of Pharmacy and Pharmaceutical Sciences, University of California, La Jolla, San Diego, CA 92093, USA; mjmeehan@health.ucsd.edu (M.M.);; 4 National Institute of Oceanography and Experimental Geophysics, 34100 Trieste, Italy; fmalfatti@units.it; 5 State Key Laboratory of Marine Environmental Science and Key Laboratory of the MOE for Coastal and Wetland Ecosystems, School of Life Sciences, Xiamen University, Xiamen 361102, China; 6 Scripps Institution of Oceanography, University of California, La Jolla, San Diego, CA 92093, USA

**Keywords:** marine algae, marine bacteria, gas chromatography–mass spectrometry (GC/MS), liquid chromatography–tandem mass spectrometry (LC-MS/MS), sea spray aerosols (SSAs)

## Abstract

Background: Glyoxal has been implicated as a significant contributor to the formation of secondary organic aerosols, which play a key role in our ability to estimate the impact of aerosols on climate. Elevated concentrations of glyoxal over open ocean waters suggest that there exists an additional source, different from urban and forest environments, which has yet to be identified. Methods: Based on mass spectrometric analyses of nascent sea spray aerosols (SSAs) and gas-phase molecules generated during the course of a controlled algal bloom, the work herein suggests that marine microorganisms are capable of excreting toluene in response to environmental stimuli. Additional culture flask experiments demonstrated that pathogenic attack could also serve as a trigger for toluene formation. Using solid-phase microextraction methods, the comparison of samples collected up-channel and over the breaking wave suggests it was transferred across the air–water interface primarily through SSA formation. Results: The presence and then absence of phenylacetic acid in the SSA days prior to the appearance of toluene support previous reports that proposed toluene is produced as a metabolite of phenylalanine through the Shikimate pathway. As a result, once in the atmosphere, toluene is susceptible to oxidation and subsequent degradation into glyoxal. Conclusions: This work adds to a minimal collection of literature that addresses the primary production of aromatic hydrocarbons from marine microorganisms and provides a potential missing source of glyoxal that should be considered when accounting for its origins in remote ocean regions.

## 1. Introduction

Toluene is a volatile organic carbon (VOC) ubiquitously found as a pollutant in the atmosphere, soil, and ground- and sea-water. Benzene, toluene, ethylbenzene, and xylene, as a group of VOCs, are termed BTEX, of which BTE are designated as priority pollutants by the Environmental Protection Agency due to their toxicity and widespread occurrence. BTEX is introduced to the environment through the combustion of petrol, jet fuel, wood products, and in cigarette smoke, as well as from the evaporation or aerosol formation of solvents, paints, degreasing agents, varnishes, adhesives, and pesticides [1]. The largest known sources for toluene are road transport and solvent use [1]. Globally, 6.9 Tg C/year of toluene is released into the environment, which represents 40% of the total BTX emissions [1].

Interest in atmospheric toluene stems from its contribution to the formation of secondary organic aerosols (SOAs), one of the greatest points of uncertainty in the ability to estimate the impact of aerosols on climate [2]. Oxidation of toluene in the presence of NO_x_ and HO_x_ species was found to yield glyoxal as the major terminal ring-opening product [3,4,5,6]. Given the highly water-soluble nature of glyoxal, it readily uptakes aqueous particles (i.e., aerosols, fog, or cloud droplets) where it can be further oxidized [7], form oligomers [8], or react with salts [9] and organics [10,11,12] to form SOAs [13,14,15,16], some of which display a range of light-absorbing properties [9,10,11,17,18,19,20]. Based on a global model of SOA formation from aromatic hydrocarbons, these species yield more SOAs when they react with hydroxyl radicals (HO^●^) in regions where the [NO]/[HO_2_] ratios are lower [1]. It has been suggested that this dependence on low- and high-NO_x_ levels relates to the competing reactions of bicyclic peroxy radical formation and cyclization, in which the former serves as the precursor for glyoxal and dominates at lower levels indicative of remote and rural atmospheres [21,22,23,24].

Part I of this two-part series addressed the discrepancies that exist within the literature of glyoxal levels measured over remote waters and demonstrated that the ocean can serve as a source of glyoxal in the atmosphere. Those findings strongly suggested that glyoxal production was linked to marine microbiological activity. The evidence provided showed that during algal senescence and decomposition over the course of a bloom, phospholipids were enriched in the sea surface microlayer (SSML), especially within the foam, and underwent radical- and ozone-initiated oxidation, thereby generating glyoxal as a degradation product. It was postulated that the enrichment of organic matter in the SSML and foam created a hydrophobic environment that affected the solubility of glyoxal such that bubble bursting from breaking wave action facilitated its transport across the air–water interface. A mechanism was proposed in which the death phase of an algal bloom could potentially serve as an important and currently missing source of glyoxal in the atmosphere.

Herein, as Part II of this series, a mechanism is proposed in which marine algae indirectly contribute to glyoxal formation through the primary production of toluene. Based on mass spectrometric analyses of nascent SSA and headspace collected during the course of a controlled phytoplankton bloom, it was demonstrated that marine toluene was produced as a biochemical response likely from algae to an environmental stressor and transferred into the atmosphere through sea spray aerosols (SSAs).

## 2. Experimental Design

### 2.1. Investigation into Marine Aerosol Chemistry and Transfer Science (IMPACTS)

**Scheme 1:**
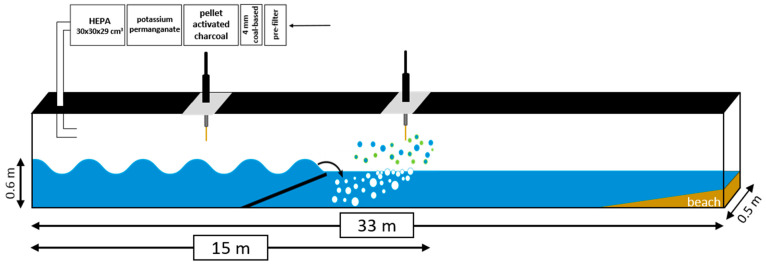
Schematic of the ocean-mimicking wave-breaking channel used during IMPACTS as it relates to SPME sampling of SSA (panel 2, over breaking wave) and gas-phase molecules (panel 1, prior to wave. The arrow represents where the air enters to be scrubbed. Aerosols are generated at 15 m formed by breaking waves; and panel 2). The wave channel is located in the Ocean-Atmosphere Facility within the Hydraulics Laboratory at Scripps Institution of Oceanography (SIO) in La Jolla, CA, USA.

A natural phytoplankton bloom was initiated on 3 July 2014 in a highly collaborative, month-long wave channel experiment, IMPACTS, Scripps Institution of Oceanography, La Jolla, Ca. [25] See Supplemental Materials Figure S1. Outside air that was scrubbed of VOCs, oxidants, and particulate matter (Scheme 1) was continually passed over the seawater surface to facilitate online sampling. Nascent SSA was generated through the action of breaking waves [25], and gas-phase molecules were sampled daily for two 2 h periods (4 h total) using solid-phase microextraction fibers (SPME; Supelco PDMS/DVB, 65 µm, Sigma 57326-U, St Louis, MI, USA) doped with 60 mg/mL *o*-phenylenediamine (*o*-PDA, Sigma P23938) dissolved in 60% tetrahydrofuran and Milli Q water (H_2_O). SSAs were also collected at ~1 L/min into an impinger containing 10 mL of H_2_O (panel 2), which is detailed in the Supplementary Materials. The SPME fibers were analyzed by gas chromatography–mass spectrometry (GC/MS Agilent 7820A, Santa Cara, CA, USA) as detailed below. The impinger samples were lyophilized and then reconstituted in 50 µL of 5% methanol solution in preparation for liquid chromatography–electrospray ionization–tandem mass spectrometry (LC-ESI-MS/MS Thermo LTQ-Orbitrap XL, Brennen, Germany) analysis. Additionally, a suite of SSA, SSML, and bulk water measurements were made daily by collaborators to monitor the physical, chemical, and biological changes that occurred throughout the course of the channel experiment.

### 2.2. Culture Flask Experiments

Cultures of 75 mL were prepared in 125 mL Erlenmeyer flasks with combinations of bacteria (*Alteromonas* sp. TW7) and green algae (*Dunaliella tertiolecta*; *D. tertiolecta*) in the appropriate media as outlined in Table 1. After inoculation, preparations were capped with rubber septa and continuously mixed by orbital gyration in ambient light conditions. Rubber septa were used to concentrate the headspace for sampling with a 5 mL air-tight syringe and analyzed by GC/MS (described in Part I). Air was filtered into the flask after each sample collection in order to renew the supply of oxygen and carbon dioxide.

### 2.3. Gas Chromatography–Mass Spectrometry (GC/MS)

An Agilent 7820A GC was used for analysis of the mass collected on the SPME fibers and headspace from the culture flask experiments, which were both manually injected into the sample port. The SPME samples were desorbed onto a Restek Corporation RTX-5 column (30 m × 250 µm × 0.25 µm; Cat# 10223) for separation before detection by the 5975 MSD system equipped with an electron impact (EI) ionization source. GC/MS parameters used for the SPME fibers were as follows: inlet = 175 °C; gradient = 90 °C (3 min), ramped to 100 °C at 0.75 °C/min (hold 2 min), and further ramped to 220 °C at 3 °C/min (hold 7 min); split = 25:1; range = *m*/*z* 45–550; EMV = 3000; MS source = 230 °C; and MS quadrupole = 150 °C. A commercially available toluene solvent was analyzed with the same GC/MS parameters; however, an autosampler was used for sample introduction in order to reproducibly inject 1 µL for 10 replicate measurements. GC/MS parameters used in the analysis of the culture flask samples were previously described [29].

### 2.4. Liquid Chromatography–Electrospray Ionization–Tandem Mass Spectrometry (LC-ESI-MS/MS)

Reconstituted SSA samples were added to glass autosampler vials with a silanized glass insert and injected onto a Phenomenex Kinetex C18 core–shell UPLC column (2.0 mm × 50 mm, 1.7 µ particle size, 100 Å pore size) using a Dionex Ultimate 3000 HPLC (Model: HPG-3000RS). The column temperature was held at 30 °C to maintain reproducible chromatography. With solvent A (0.1% acetic acid in LC/MS-grade H_2_O) and solvent B (0.1% acetic acid in LC/MS-grade acetonitrile), the samples were separated at 0.5 mL/min using the following gradient: 5% B (hold 1 min), ramp to 80% B over 7 min, and to 100% B in 1 min (hold 0.5 min), before re-equilibrating the column at 5% B for 1 min. The column eluent was coupled to a high-resolution Bruker Daltonics Maxis Impact quadrupole time-of-flight (qTOF) mass spectrometer. All samples were acquired in positive-ion mode using the following parameters: electrospray voltage = 3.5 kV; nebulizer pressure = 2 bar; N_2_ drying gas = 9 L/min @ 200 °C; dual ion funnel RF = 250 V (peak-to-peak, pp); transfer hexapole RF = 70 V pp; isolation quadrupole energy = 5 eV; MS collision cell energy = 10 eV; MS scan rate = 3 Hz; and MS^2^ variable scan rate = 7–10 Hz. Each MS scan was followed by 5 subsequent MS^2^ scans. Precursor ions were selected to be fragmented in a data-dependent manner in which the 5 most abundant ions in each MS scan were fragmented. Ions were allowed to be fragmented 3 times before being added to an exclusion list in order to maximize the potential to acquire quality MS^2^ spectra. MS^2^ collision energy stepping was used to allow a sampling of 4 separate collision energies for each molecule ranging from 18 to 52 eV. The 4 energies used per each molecule varied depending on its mass-to-charge ratio (*m*/*z*). The collision RF (post-collision refocusing) was also stepped in order to optimally trap the maximum number of fragment ions. An internal calibrant was constantly introduced into the mass spectrometer along with the sampler to allow re-calibration of the resulting data in order to counter drifts in mass accuracy of the mass spectrometer.

### 2.5. Chlorophyll a Measurements

Chlorophyll *a* concentrations of the bulk seawater were measured fluorometrically using a Wetlabs ECO BBFL2 sensor (470/695 nm EX/EM) and Turner AquaFluor handheld unit (395,130/≥600 nm EX/EM). The Wetlabs sensor was integrated into a flow-through system with a pump, housing, and additional sensors for continuous measurements; the AquaFluor was used with discrete samples. The Wetlabs sensor was calibrated at the factory against *T. weissflogii* cultures and used as received. Measurements made with the AquaFluor were calibrated against simultaneous values recorded by the Wetlabs sensor, and the data from both instruments were combined into a single continuous time series.

### 2.6. Ammonium, Nitrate, and Phosphate (Nutrients) Measurements

All nutrient analyses were performed on a SEAL Analytical segmented continuous-flow system located in the Ocean Data Facility (ODF) at SIO in La Jolla, CA, USA. After each run, strip charts were reviewed for any problems, and final concentrations (micromoles per liter) were calculated using SEAL Analytical AACE software, version 6.07. Nitrate and ammonium concentrations were determined using modified procedures presented by Armstrong et al. (1967) [30] and Koroleff et al. (1969) [31], respectively. Detailed procedures are available on the ODF website under the documentations link (https://scripps.ucsd.edu/ships/shipboard-technical-support/odf, accessed on 15 June 2014).

### 2.7. Ectohydrolytic Chitinase (β-1,4-ploy-N-acetylglucosaminidase) Activity Measurements

The fluorogenic 4-methyl-umbelliferone-N-acetyl-β-D-glucosaminide (Sigma-Aldrich Cat # 69585, St. Louis, MO, USA) substrate analog was used at a saturating concentration (20 µM) to quantify chitinase ectohydrolytic activities using MUF-N-acetyl-β-D-glucosamine [32,33]. Assays were performed in 96-well black plates incubated in the dark at in situ temperature for 1 h. Fluorescence was measured immediately after substrate addition and again at the end of the incubation [34] at 355/460 nm (excitation/emission) using the multiplate reader fluorometer SpectraMax M3 (Molecular Device, San Jose, CA, USA). The difference between the blank-corrected (three blanks) fluorescence and the average of 3 replicates was divided by the time of incubation and calibrated against the 4-MUF standard. Chitinase activities were computed as nmol of substrate hydrolyzed L^−1^ h^−1^ in bulk and SSML samples separately, and the measurements were combined into one data point.

## 3. Results and Discussion

### 3.1. Investigation into Marine Aerosol Chemistry and Transfer Science (IMPACTS)

**Figure 1:**
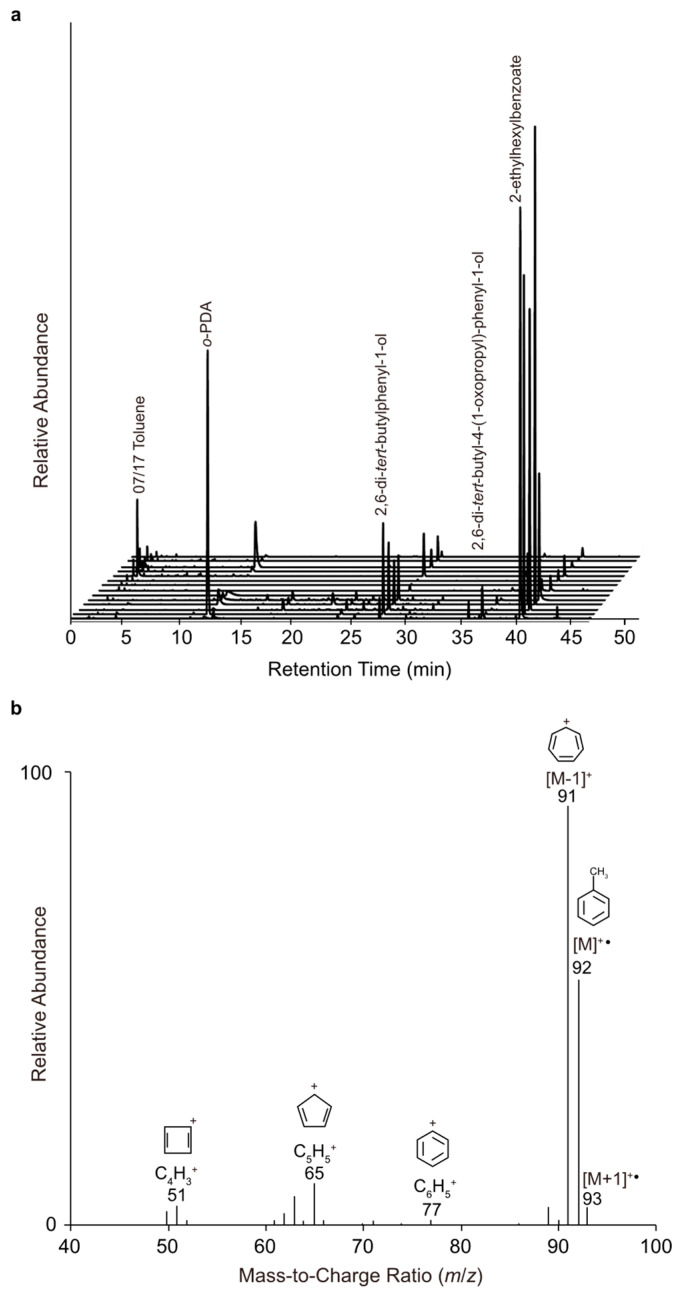
Temporal plot of GC total ion chromatograms of the mass collected on o-PDA-doped SPME fibers from panel 2 (see Experimental Design) during IMPACTS (**a**). Annotated EI-MS spectrum of toluene (RT 2.1 min) observed on 07/17/2014 (**b**). All retention times were confirmed with authentic standards.

To determine whether a marine source of toluene exists as a result of microbiological activity, a mesocom experiment, Investigation into Marine Aerosol Particle Chemistry and Transfer Science (IMPACTS), was performed in a wave channel filled with 13,000 L of seawater pumped from the pier at Scripps Institution of Oceanography (SIO) in La Jolla, CA, USA. The atmosphere within the wave channel was sampled prior to (panel 1) and over (panel 2) a breaking wave using SPME fibers doped with *o*-PDA (Scheme 1). Separate sampling locations were used in order to distinguish between molecules transferred primarily in the gas phase (panel 1) and those in SSA (panel 2). The collected mass was desorbed onto a GC column and analyzed using MS. As evident by the time course of GC traces in Figure 1a, the addition of *o*-PDA increased van der Waals interactions between the fiber and phenyl-containing molecules [i.e., toluene (RT 2 min); *o*-PDA dopant (RT 10 min); 2,6-di-*tert*-butylphenyl-1-ol (RT 27 min); 2,6-di-*tert*-butyl-4-methylphenyl-1-ol (RT 31 min); benzophenone (RT 35 min); 2,6-di-*tert*-butyl-4-(1-oxopropyl)-phenyl-1-ol (RT 37 min); 2-ethylhexylbenzoate (RT 39 min)].

**Figure 2:**
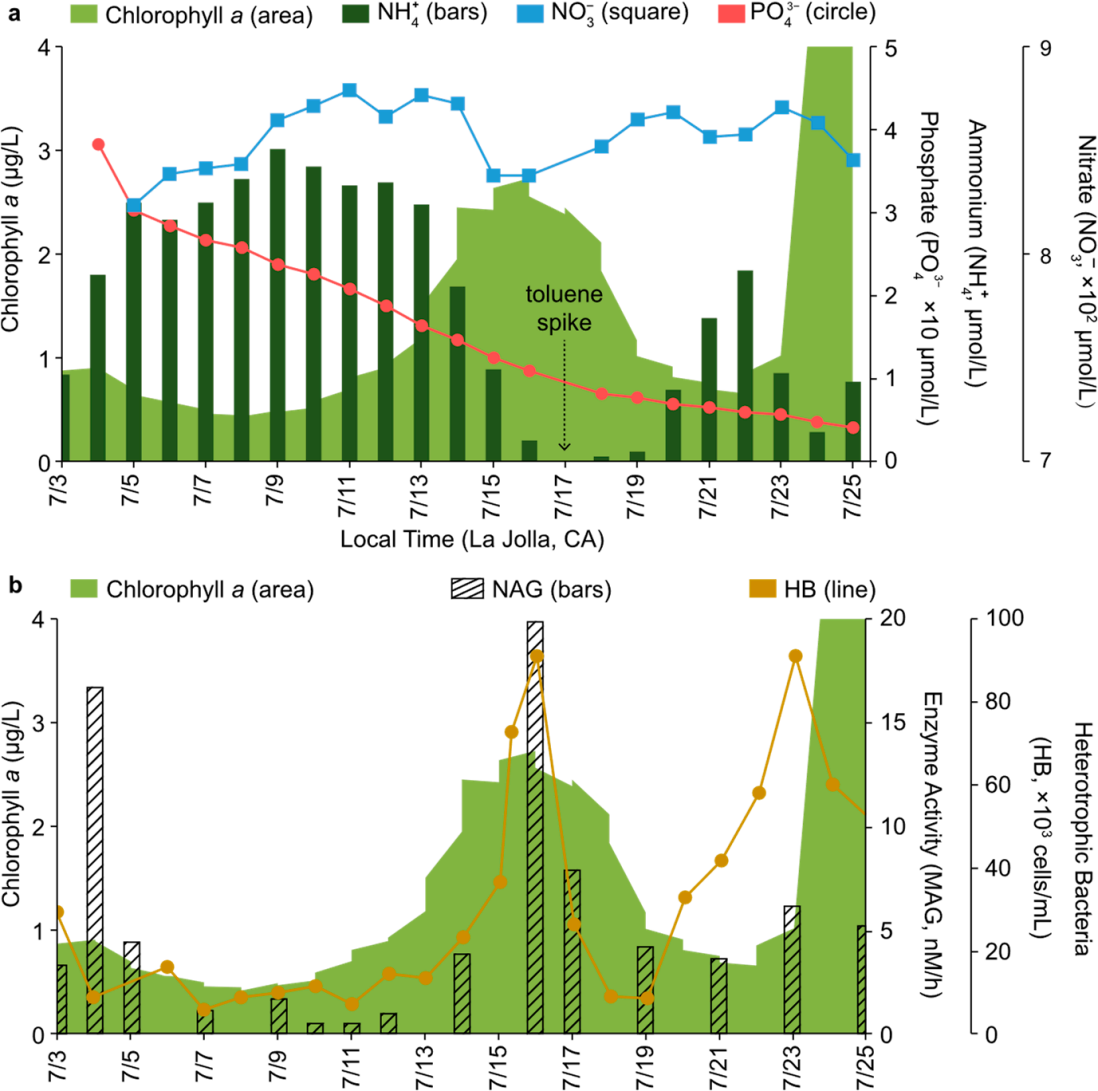
Ammonium (NH_4_^+^, bars, μmol/L), nitrate (NO_3−_, squares, μmol/L), and phosphate (PO_4_^3−^, circles, μmol/L) (**a**) heterotrophic bacteria (HB, line, cells/mL) and chitinase activity (NAG, nM/h, bars) (**b**) plotted against chlorophyll a levels (area, μg/L) during the course of IMPACTS.

As shown in Figure 1, there was a sudden spike in toluene emissions on 17 July 2014 (Day 15), which correlates with the peak in chlorophyll *a* (Figure 2). This toluene was detected on SPME fibers from panel 2, but not panel 1, suggesting its transfer across the air–water interface was facilitated primarily by SSA formation. Given that the atmospheric (outside) air drawn into the wave channel to aid in online sampling was scrubbed of oxidants before its delivery, it was not possible for the transferred toluene to be converted into glyoxal as previously described and then quantified [6]. For context, with respect to the concentration of observed toluene on 17 July 2014, continuous online sampling of SSA and gas-phase molecules throughout IMPACTS was conducted by aerosol time-of-flight mass spectrometry (ATOFMS) and chemical ionization time-of-flight mass spectrometry (CITOFMS), respectively. A signal corresponding to the likely off-gassing of anthropogenic toluene (pollution) was just at or below the detection limit of both the ATOFMS and CITOFMS, as evident by a combination of positive and negative integration values (https://doi.org/10.6075/J0J966QP). Aerosols were also collected into an impinger of deionized water and analyzed by LC-ESI-MS/MS; however, this technique is not suitable for toluene detection. Additionally, whole air was concentrated into evacuated canisters at discrete time points during IMPACTS and analyzed by GC/MS. The combined BTEX concentrations per sample ranged from 0.75 to 1.3 ppmv (https://doi.org/10.6075/J0J966QP). These levels were too low for characterization using the SPME-GC/MS method detailed herein. Taking into consideration the minimal toluene signal with the ATOFMS, CITOFMS, and whole air measurements, along with the peak intensities of the sunscreen- (RT 39 min), plastic- (RT 35 min), and fuel-additives (RT 27, 31, and 37 min) detected by SPME-GC/MS (Figure 1a), it follows that the toluene emissions on 17 July 2014 were well above background pollution levels.

Figure 2a plots the ammonium (NH_4_^+^) and nitrate (NO_3−_) levels relative to the concentration of chlorophyll *a* throughout the mesocom experiment. The strong inverse relationship between NH_4_^+^ and chlorophyll *a* is consistent with the belief that NH_4_^+^, opposed to NO_3_^−^, is the preferred algal source for nitrogen uptake. This is likely due to the fact that it is more energetically costly to reduce the oxyanion to the correct oxidation state for incorporation into amines and amides [35]. The recovery of NH_4_^+^ levels upon the declining chlorophyll *a* concentration is consistent with its production by bacteria and archaea and its assimilation by phytoplankton and bacteria, resulting in a rapid turnover in the available pool of NH_4_^+^ in near-surface water conditions [36,37,38]. As NH_4_^+^ was depleted below 1 µM, the NO_3_^−^ concentration dropped, and toluene was detected 2 days later. This ammonium–nitrate relationship is consistent with reports that NH_4_^+^ concentrations in excess of 1 µM inhibit, to some degree, NO_3_^−^ uptake in a shared environment [35]. Chamber experiments conducted by Hieden et al. (1999) reported a similar trend of toluene emissions from sunflowers and pine [39]. Specifically, the release of toluene from sunflowers increased in the complete absence of nitrogen (N; ~1 day after N-depletion) and wound infliction (instantaneously) and from pine when its needles were under pathogenic attack. The change in toluene flux was attributed to the stress response of the plants. In the same vein, the spike in toluene emissions observed during IMPACTS may have originated from the phytoplankton in response to the change in NH_4_^+^ levels that appear to have stimulated NO_3_^-^ uptake. If N limitations were involved in the primary production of toluene during IMPACTS, it should be noted that environmental factors [i.e., peak in the heterotrophic bacteria (HB) concentration on 16 July 2014, Figure 2b] and the availability of other nutrients [i.e., depleting phosphate (P) concentrations, Figure 2a] likely worked in concert as algal stressors to promote this response.

The spike in HB, which coincided with the peak in chlorophyll *a* (Figure 2b), presents an alternative theory that the observed toluene may have had prokaryotic origins. As shown in Figure 2b, there was a corresponding increase in chitinase activity on 16 July 2014. Chitinase is a lysosomal enzyme with specificity towards β-1,4-ploy-*N*-acetylglucosamine (NAG), which is a component of a more complex peptidoglycan structure found in bacterial cell walls, as well as other organisms. Chitinase is one in a group of ectoenzymes (i.e., phosphatase, lipase, nuclease, β-*N*-acetylhexosaminidase, and α-/β-glucosidase) largely responsible for the degradation of dissolved organic matter (DOM) in aquatic environments [33,40,41,42,43,44]. Although its activity is typically associated with bacterial and cyanobacterial DOM recycling, it has been demonstrated in both cultured and field experiments that various taxa of eukaryotic marine phytoplankton also possess this enzyme [45]. In some instances, it was found that the β-1,4-ploy-*N*-acetylglucosaminidase activity was greatest in N- and/or P-depleted cultures [45]. Thus, Gram-positive HB would be highly susceptible to hydrolysis by algal β-1,4-ploy-*N*-acetylglucosaminidase, which may have been exacerbated by the declining availability of key nutrients. Given the correlation between the peak in HB and chlorophyll *a*, and a precedence for bacterial involvement in biogenic toluene [46,47,48,49], it is quite plausible that the chitinase activity of the phytoplankton may have served as a trigger for prokaryotic-derived toluene.

### 3.2. Culture Flask Studies

**Figure 3:**
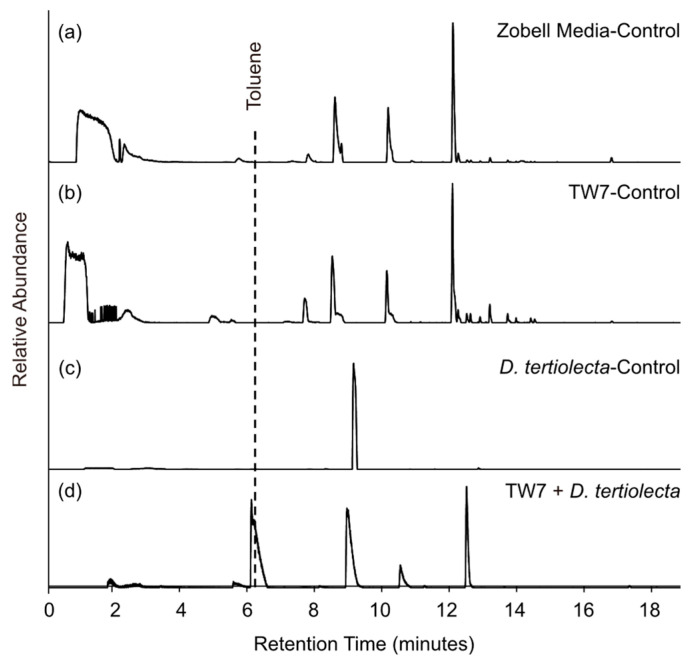
GC/MS total ion chromatograms of the headspace from the Zobell media control (**a**), TW7 control (**b**), *D. teriolecta* control (**c**), and TW7 + *D. teriolecta* (**d**) culture flasks collected using an air-tight syringe.

To confirm that toluene can be produced biochemically from marine organisms, *Dunaliella tertiolecta* (*D. tertiolecta*), a green algae common to marine waters, was cultured in a flask without the addition of raw seawater in both the absence and presence of the bacteria *Alteromonas* TW7 (TW7). As shown in Figure 3, toluene was readily detected in the TW7 + *D. tertiolecta* (d) culture less than 24 h after inoculation. The absence of toluene in the Zobell media (a), TW7 (b), and *D. tertiolecta* (c) control cultures suggests its production is linked to a response from the algae initiated by the introduction of a potential pathogen, in this case, TW7. Alternatively, the toluene may have originated from the bacteria based on a handful of reports that have tied its production to prokaryotic activity in low-oxygen environments, which was representative of the culture flask experiments, but not IMPACTS [46,47,48,49]. In either case, these findings clearly demonstrate that toluene can be formed as a product of microbiological activity.

### 3.3. ^13^C Isotopic Fractionation

The ^13^C/^12^C ratio of toluene detected during IMPACTS was compared to the TW7 + *D. tertiolecta* culture and a commercially purchased solvent in order to determine whether it was indeed the result of primary production, merely the transfer of anthropogenic pollution, or perhaps both. The ^13^C/^12^C ratio was calculated based on the non-corrected relative peak intensities of the [M + 1]^+●^/[M]^+●^ ions that were generated during GC/MS analysis (Figure 1b). The toluene from IMPACTS (^13^C/^12^C = 0.010559) and the TW7 + *D. tertiolecta* culture (^13^C/^12^C = 0.010667) were significantly enriched in ^13^C content relative to the commercial solvent (^13^C/^12^C = 0.010274 ± 0.89%, 10 replicates). These findings suggest the observed toluene during IMPACTS and in the TW7 + *D. tertiolecta* culture were from a source different than petroleum. The disparity in ^13^C/^12^C content between petroleum- and non-petroleum-derived organics stems from the initial carbon source. It has been shown that lipids (i.e., fatty acids, fatty acid esters, hydrocarbons, terpenoids, steroids, and alkaloids) are the primary precursors of petroleum products (terrestrial- and marine-based) [50,51,52,53], which are consistently depleted in ^13^C content relative to the remainder of the host organism [54,55,56,57]. Given that low-molecular-weight aromatic hydrocarbons present in petroleum come from the chemical decomposition of terpenoids or the disproportionation reactions involving isoprenoids [52], it follows that the commercially available toluene would also be depleted of ^13^C. ^13^C fractionation of microbiologically derived organics from photosynthetic organisms is governed by the ribulose-1,5-bisphosphate carboxylase/oxygenase (Rubisco) enzyme [58]. Rubisco is responsible for the fixation of atmospheric carbon dioxide (CO_2_, ^13^C/^12^C = 0.011147) as the rate-limiting step in the Calvin cycle. It has been shown that Rubisco preferentially incorporates ^12^CO_2_, thus resulting in ^13^C depletion of ^13^C/^12^C ~0.000303 [59]. In marine organisms such as microalgae, Rubisco can also utilize dissolved hydrogen carbonate (HCO_3_^−^, ^13^C/^12^C = 0.0112372), which is reduced to CO_2_ by carbonic anhydrase before fixation [60]. It has been demonstrated that N limitations as well as the N source have a considerable effect on the ^13^C discrimination of Rubisco by promoting a CO_2_-concentrating mechanism (CCM) that is found in a wide range of marine algae, including *D. tertiolecta* [61,62,63,64]. Briefly, CCM activity saturates the active site of Rubisco with CO_2_ at concentrations far greater than available in the surrounding environment, implying that previously discriminated ^13^CO_2_ is also incorporated by this mechanism [65]. In an NH_4_^+^-limited environment, algae will assimilate NO_3_^−^ [35], which requires four times more energy to process and subsequently triggers CCM activity [61,66]. Although cells grow as well, if not better, utilizing NO_3_^−^ compared to NH_4_^+^, it has been shown that activating CCM lowers the affinity for algal uptake of dissolved inorganic carbon (i.e., CO_2_ and HCO_3_^−^), thereby creating a “closed system” in which the inherent ^13^C discrimination by Rubisco is no longer observed [61,66]. This creates a decrease in isotopic fractionation and thus a larger ^13^C/^12^C ratio. The difference in ^13^C content between IMPACTS and the TW7 + *D. tertiolecta* culture may reflect this phenomenon. As previously discussed, both NH_4_^+^ and NO_3_^−^ were available for uptake during IMPACTS; however, only NO_3_^−^ was supplied in the culture experiment, suggesting CCM may have been activated soon after inoculation, and thus more ^13^C was incorporated. Given that CCM is an active process that requires energy (i.e., adenosine triphosphate, ATP), carbon assimilation by this process is also affected by the availability of inorganic phosphorous [67,68]. It was reported that in a P-limited environment, CCM activity is down-regulated, which results in greater ^13^C discrimination [67,68]. This too may explain the smaller ^13^C/^12^C ratio of toluene from IMPACTS relative to the culture flask that was grown in repleted media. The exact interplay between CCM activity, changes in nutrient sources and availability during the growth cycle, and the subsequent ^13^C/^12^C ratio has not been studied in depth. It is also plausible that the slightly less ^13^C-enriched toluene detected during IMPACTS (^13^C/^12^C = 0.010559) compared to the TW7 + *D. tertiolecta* culture (^13^C/^12^C = 0.010667) may represent a mixture of petroleum-derived and microbiologically derived toluene. Nevertheless, it is evident that the toluene detected during IMPACTS and the cultured experiment contained statistically more ^13^C content than the commercial solvent, which is consistent with having biochemical origins, not simply petroleum, which may be linked to the stress response of the microorganisms.

### 3.4. Biochemical Pathway for Toluene Formation

**Figure 4:**
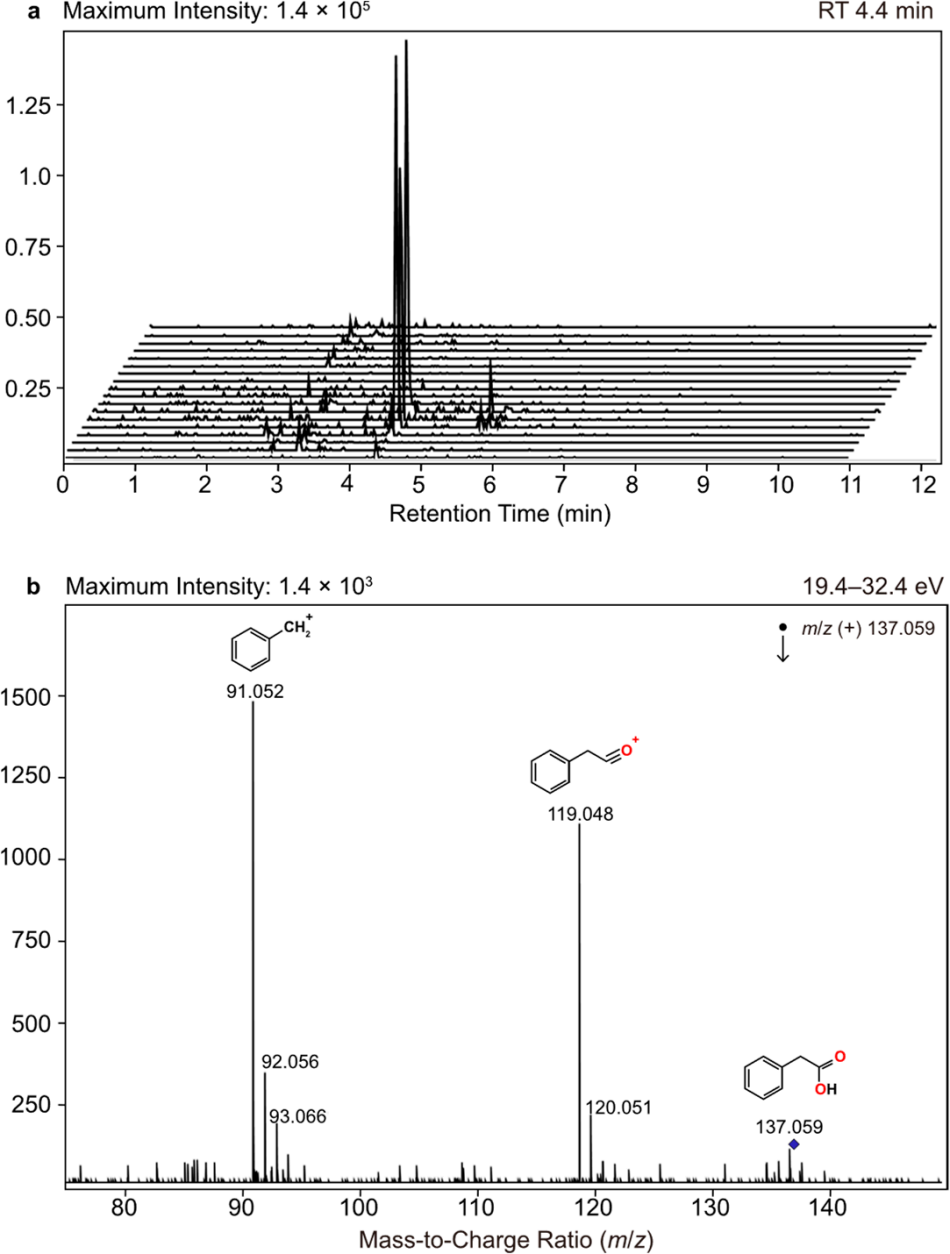
(**a**) Temporal plot of selected ion chromatograms (*m*/*z* 137.059) from sea spray aerosols collected via impinger. (**b**) Annotated positive-ion mode ESI-MS/MS spectrum of *m*/*z* 137.059 (RT 4.4 min), which corresponds to phenylacetic acid.

It has been indirectly demonstrated through stable isotope labeling experiments that toluene is produced biochemically through the Shikimate pathway, likely from phenylacetate, which is a metabolite of phenylalanine [46,47,49]. In higher plants and algae, phenylacetate has been identified as an auxin-like substance, which is a growth-promoting hormone found in roots, shoots, pollen, and exudates [69,70,71]. In insects such as ants, it is released by the metapleural glands as an antimicrobial defense [72]. It has also been identified as a product of various bacteroides found in human oral clinical isolates [73]. Herein, phenylacetic acid was characterized during 7–10 July 2014 by LC-ESI-MS/MS (Figure 4) in SSA samples collected via impinger, which appears to correspond with the end stage of the lag phase of the algal growth prior to toluene detection. 4-hydroxyphenylacetic acid, which is a metabolite of tyrosine formed in the Shikimate pathway, was also characterized in the SSA during this same time frame. Similarly, 4-hydroxyphenylacetic acid exhibits growth-promoting activity in marine algae; however, its reported abundance was four times less than phenylacetic acid [69], which explains why the LC-ESI-MS/MS signal was near the limit of detection, and its corresponding reduction product, 4-hydroxytoluene, was not observed, which would have provided additional support for this proposed mechanism. More systematic labeling experiments are needed to determine the exact mechanism of formation and to gain a better understanding of the conditions that promote toluene production.

## 4. Conclusions

This work demonstrated that toluene can be formed biogenically from marine microbiological activity and transferred into the atmosphere through SSA formation. Evidence suggested that its formation was linked to a stress response in the microorganisms that may have been triggered by a change in nutrient levels and/or source, as well as the presence of potential pathogens. These findings support a handful of studies that showed toluene can be formed from terrestrial plants under duress [39] and aquatic bacteria in anoxic environments [46,47,48,49]. The work herein represents the first findings that marine algae may also participate in this chemistry. At present, it is unclear what concentrations of toluene could be produced by this mechanism and how those levels would compare to other sources in non-urban atmospheres. Although reports of marine toluene are sparse within the literature, and its flux into the atmosphere is currently unknown, the knowledge of its primary production should be considered when attempting to close the gap between observed glyoxal concentrations in remote environments and known sources of its formation.

## Figures and Tables

**Table 1:** Preparations for culture flask experiments.

**Experiments**	**ZoBell** **[26]** **Medium**	**mHSM** **[27,28]** **Medium**	**Raw Seawater**	***Alteromonas* sp. TW7**	***Dunaliella tertiolecta***	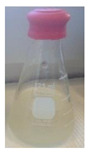 Culture flask.
ZoBell control	75 mL					
TW7 control	73 mL			2 mL		
*D. tert* control		74 mL			1 mL	
TW7 + *D. tertiolecta*	36 mL	36 mL		2 mL	1 mL	

## Data Availability

https://doi.org/10.6075/J0J966QP.

## Supplementary Materials

The following supporting information can be downloaded at: https://www.mdpi.com/article/10.3390/metabo14110631/s1, Figure S1: NIST Mass Spectra of quinoxaline; Figure S2: Schematic of the impinger set-up used within the wave channel during IMPACTS; Figure S3: Phytoplankton bloom conditions during IMPACTS.
