# Supplementary material for: Possible Missing Sources of Atmospheric Glyoxal Part II: Oxidation of Toluene Derived from the Primary Production of Marine Microorganisms
Source: Metabolites. 2024 Nov 16;14(11):631. doi: 10.3390/metabo14110631 (PMC11596052; doi:10.3390/metabo14110631)
Supplement: Supplementary file 1 [file metabolites-14-00631-s001.zip › metabolites-3263085-supplementary.pdf]

Supporting Information

Possible Missing Sources of Atmospheric Glyoxal Part II: Oxidation of Toluene Derived from the Primary Production of Marine Microorganisms

Renee T. Williams [1], Annika Caspers-Brown [2], Jennifer Michaud [2], Natalie Stevens [2], Michael Meehan [3], Camille M. Sultana [2], Christopher Lee [2], Francesca Malfatti [4], Yanyan Zhou [5], Farooq Azam [6], Kimberly A. Prather [2,6], Pieter Dorrestein [3], Michael D. Burkart [2] and Robert S Pomeroy [2,*]

1 Williams Biotech Consulting San Bruno, CA 90466, USA; rw@ags-tx.com
2 Department of Chemistry and Biochemistry, University of California, La Jolla, San Diego, CA 92093, USA; annika.brown@hamiltoncompany.com (A.C.-B.); jennifer.michaud@scalebiosciences.com (J.M.); natalie.stevens@catalent.com (N.S.); camille.sultana@arb.ca.gov (C.M.S.); chl257@ucsd.edu (C.L.); kprather@ucsd.edu (K.A.P.); mburkart@ucsd.edu (M.B.)
3 Skaggs School of Pharmacy and Pharmaceutical Sciences, University of California, La Jolla, San Diego, CA 92093, USA; mjmeehan@health.ucsd.edu (M.M.); pdorrestein@health.ucsd.edu (P.D.)
4 National Institute of Oceanography and Experimental Geophysics, 34100 Trieste, Italy; fmalfatti@units.it
5 State Key Laboratory of Marine Environmental Science and Key Laboratory of the MOE for Coastal and Wetland Ecosystems, School of Life Sciences, Xiamen University, Xiamen 361102, China; zhouyyahgy@scsio.ac.cn
6 Scripps Institution of Oceanography, University of California, La Jolla, San Diego, CA 92093, USA; fazam@ucsd.edu
* Correspondence: rpomeroy@ucsd.edu; Tel.: +1-858-822-5736

Discrepancies in Glyoxal Measurement (Background from Part I)

Glyoxal, the smallest of α-dicarbonyl compounds, is a ubiquitous gas-phase oxidation product of a variety of volatile organic compounds (VOCs) found in the atmosphere. The vast majority of research has focused on elevated glyoxal concentrations in urban environments as a result of vehicle emissions, in addition to other anthropogenic sources including biomass and fossil fuel burning [1–8]. The largest known precursor for glyoxal is isoprene, which is emitted from terrestrial biogenic sources, and to a much lesser extent, from the oxidation of monoterpenes [9–13]. Interest in the origins and levels of glyoxal within the atmosphere lies in its potential contribution to aerosol particles that serve as cloud condensation nuclei, which is one of the most poorly understood topics in atmospheric aerosol research [14]. Given the highly water soluble nature of glyoxal, it readily uptakes water to form aqueous particles (i.e., aerosols, fog, or cloud droplets) where it can be oxidized [15], form oligomers [16], and react with salts [17] and organics [18–20] to form secondary organic aerosols (SOA) [9,21–23]. It has been shown that

sulfuric acid-catalyzed heterogeneous reactions involving glyoxal, as well as other aldehydes, can lead to the growth of newly nucleated nanoparticles [14,24–26]. Moreover, aqueous phase processing of glyoxal can result in a range of light

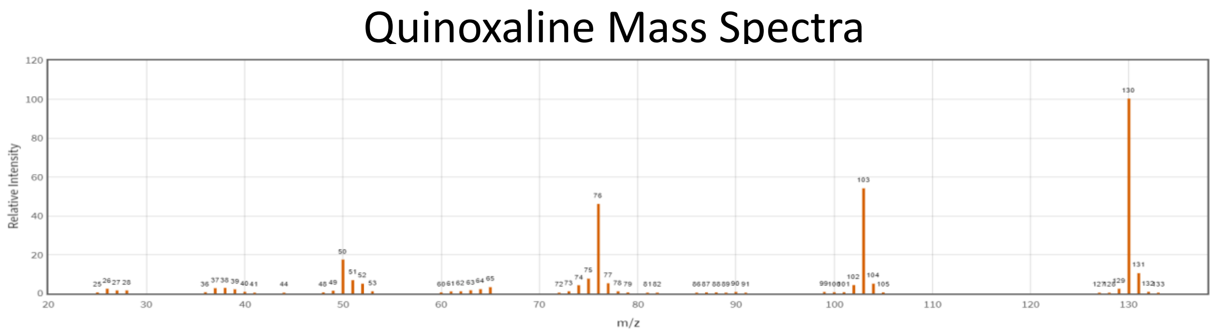

Figure S1. NIST Mass Spectra of quinoxaline

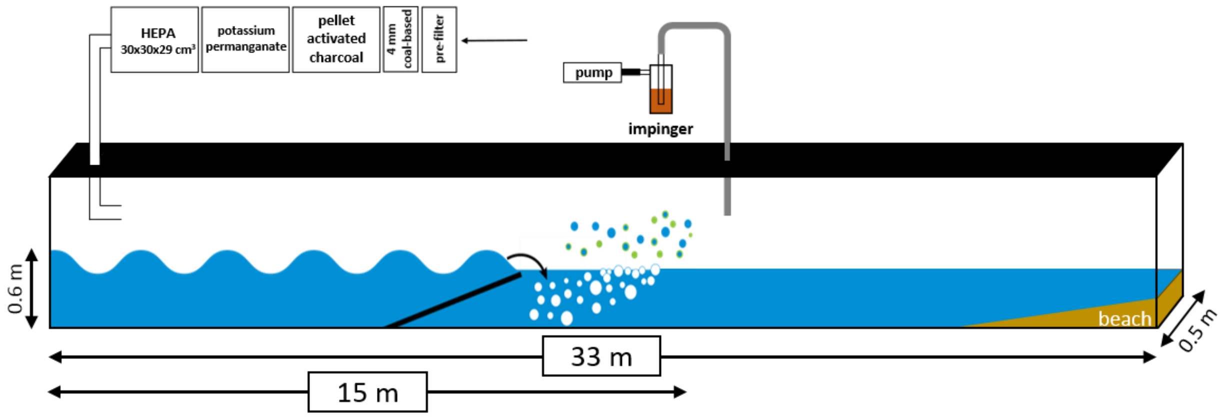

Figure S2. Schematic of the impinger set-up used within the wave channel during IMPACTS.

Chlorophyll a as Measured by Fluorescence versus Day

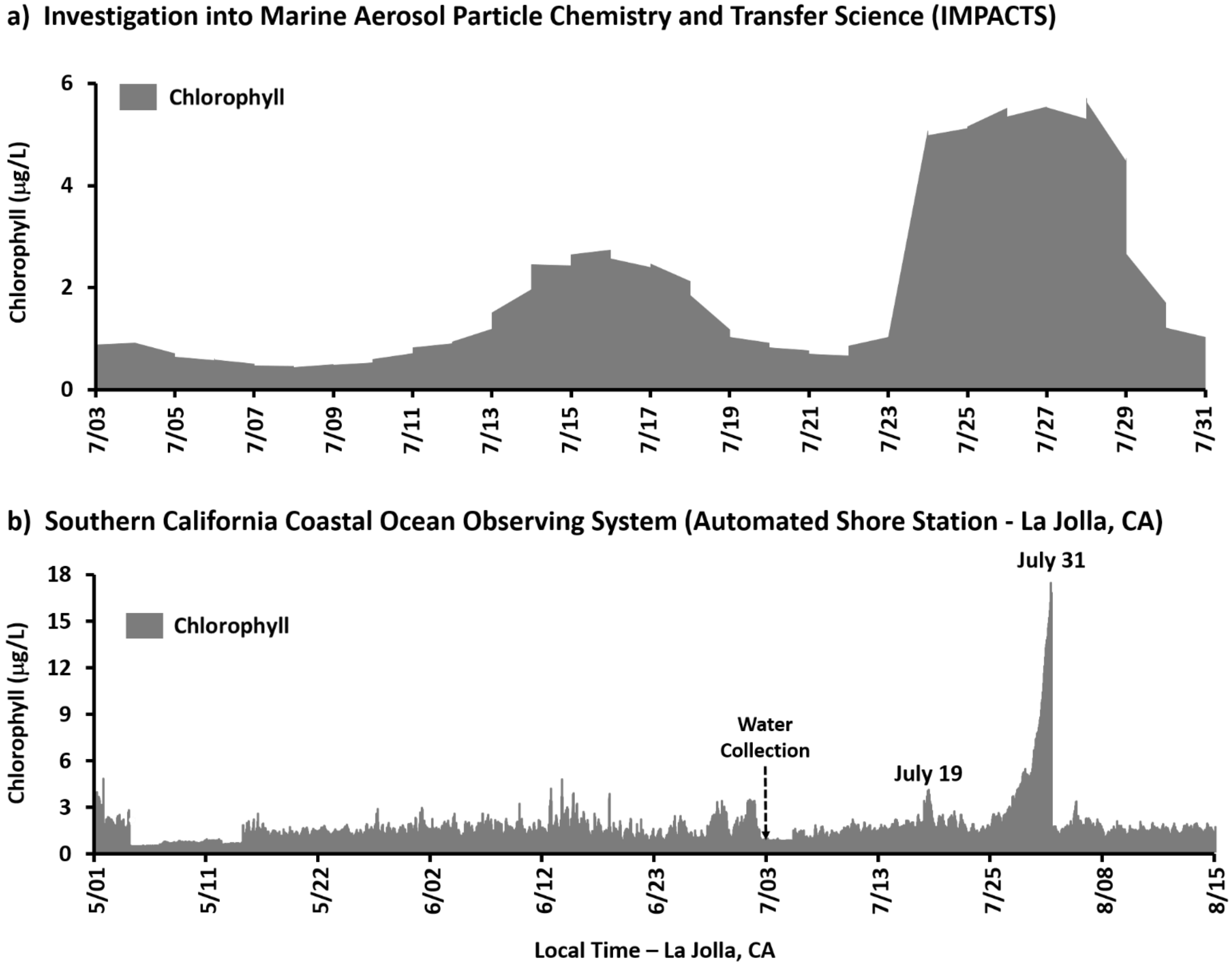

Figure S3. Phytoplankton bloom conditions during IMPACTS (a), and in the coastal waters off of the SIO pier in La Jolla, CA (b)
